# Vertebrate Hosts as Islands: Dynamics of Selection, Immigration, Loss, Persistence, and Potential Function of Bacteria on Salamander Skin

**DOI:** 10.3389/fmicb.2016.00333

**Published:** 2016-03-16

**Authors:** Andrew H. Loudon, Arvind Venkataraman, William Van Treuren, Douglas C. Woodhams, Laura Wegener Parfrey, Valerie J. McKenzie, Rob Knight, Thomas M. Schmidt, Reid N. Harris

**Affiliations:** ^1^Department of Biology, James Madison University, HarrisonburgVA, USA; ^2^Department of Internal Medicine, University of Michigan, Ann ArborMI, USA; ^3^BioFrontiers Institute, University of Colorado, BoulderCO, USA; ^4^Department of Ecology and Evolutionary Biology, University of Colorado, BoulderCO, USA

**Keywords:** neutral model, host-associated microbial communities, Island biogeography, *Plethodon cinereus*, *Batrachochytrium dendrobatidis*, symbiosis, antifungal

## Abstract

Skin bacterial communities can protect amphibians from a fungal pathogen; however, little is known about how these communities are maintained. We used a neutral model of community ecology to identify bacteria that are maintained on salamanders by selection or by dispersal from a bacterial reservoir (soil) and ecological drift. We found that 75% (9/12) of bacteria that were consistent with positive selection, <1% of bacteria that were consistent with random dispersal and none of the bacteria that were consistent under negative selection had a 97% or greater match to antifungal isolates. Additionally we performed an experiment where salamanders were either provided or denied a bacterial reservoir and estimated immigration and loss (emigration and local extinction) rates of bacteria on salamanders in both treatments. Loss was strongly related to bacterial richness, suggesting competition is important for structuring the community. Bacteria closely related to antifungal isolates were more likely to persist on salamanders with or without a bacterial reservoir, suggesting they had a competitive advantage. Furthermore, over-represented and under-represented operational taxonomic units (OTUs) had similar persistence on salamanders when a bacterial reservoir was present. However, under-represented OTUs were less likely to persist in the absence of a bacterial reservoir, suggesting that the over-represented and under-represented bacteria were selected against or for on salamanders through time. Our findings from the neutral model, migration and persistence analyses show that bacteria that exhibit a high similarity to antifungal isolates persist on salamanders, which likely protect hosts against pathogens and improve fitness. This research is one of the first to apply ecological theory to investigate assembly of host associated-bacterial communities, which can provide insights for probiotic bioaugmentation as a conservation strategy against disease.

## Introduction

Amphibians are susceptible to the fungal pathogen *Batrachochytrium dendrobatidis*, which infects the skin and causes the disease chytridiomycosis (Berger et al., 1998). Bacterial communities on the surfaces of amphibians’ skin can protect against the pathogen (Becker and Harris, 2010; Bletz et al., 2013). In one host species, the composition of bacterial communities on the skin before infection was associated with mortality caused by chytridiomycosis (Becker et al., 2015). Within these communities, specific bacteria are likely responsible for disease resistance. For example, the bacterium *Janthinobacterium lividum* has been administered to amphibians as a probiotic and confers disease resistance (Becker et al., 2009; Harris et al., 2009). This bacterium inhibits *B. dendrobatidis* by producing antifungal metabolites such as violacein (Brucker et al., 2008). Indeed, red-backed salamanders (*Plethodon cinereus*) with a greater abundance of *J. lividum* had greater violacein concentrations and concomitantly experienced less mortality from *B. dendrobatidis* (Becker et al., 2009). Other specific bacteria found on amphibians, such as members of Pseudomonadaceae, Flavobacteriaceae and Comamonadaceae, likely protect amphibians from disease (Becker et al., 2015). This work aims to elucidate how these common bacteria are maintained as a community on salamanders.

Red-backed salamanders obtain their skin bacteria through the environment (Muletz et al., 2012), and the availability of bacteria in the environment greatly influences the salamanders’ skin bacterial community (Loudon et al., 2014b). Though skin bacteria are acquired from the environment (e.g., soil, rocks), skin communities remain distinct because the abundance of many bacterial taxa differs on salamanders compared to their environment. Furthermore, without a bacterial reservoir (soil), salamander skin bacterial communities lost diversity and became uneven with just a few taxa dominating the community (Loudon et al., 2014b). Therefore, evidence strongly suggests that the maintenance of bacterial communities on salamander skins is influenced by continual contact between the host and bacteria in the environment.

Vellend’s synthesis in community ecology provides a framework for discussing the processes that determine bacterial community maintenance on vertebrate skins (Vellend, 2010; Nemergut et al., 2013). Vellend’s synthesis places the processes that shape communities into four categories: selection, drift, dispersal, and speciation. We focus on selection, drift and dispersal since speciation is likely not relevant in our short-term study, which took place over 28 days.

Selective processes include ecological interactions amongst microbial species and positive or negative selection by the host, which are processes that favor one species over the other. Neutral processes are equally likely to influence all species. Such processes include random dispersal of bacteria between salamander skins and a bacterial reservoir (soil), as well as random birth and death processes (ecological drift). In other words, there is an equal opportunity for all microbes to disperse from soil to salamander skins, grow there and be lost or removed from salamander skins.

It is now recognized that both neutral and selective processes typically influence community composition (Vellend, 2010). However, the relative importance of these two processes is contingent upon the study system and is context dependent. We determined the relative importance of selective and neutral processes in maintaining the salamander’s skin bacterial community by using a neutral model of community ecology (Morris et al., 2013; Venkataraman et al., 2015). The neutral model assumes that the composition of a community can be explained solely via the neutral processes of random dispersal of community members from the surroundings and ecological drift. Therefore, the neutral model serves as a valuable null hypothesis to gain insight into the taxa that are present as a result of selective rather than neutral processes. For species that are consistent with the model expectations, we cannot reject the possibility that they are being detected because of random dispersal and ecological drift. Species that deviate from the model expectations are the strongest candidates for undergoing selection (both positive and negative). We considered the soil as a potential source of microbes for salamander skin. The relative abundance of an operational taxonomic unit (OTU) in the soil is used to calculate the probability of detecting that OTU in salamander skins because of dispersal and ecological drift. With this approach, OTUs fall into three categories: (i) those that are consistent with the model expectations – neutrally-distributed on salamander skins, (ii) those that positively deviate from the model expectations – over-represented on salamander skins, and (iii) those that negatively deviate from the model expectations – under-represented on salamander skins.

Previously we found that presence of a natural bacterial reservoir was important for the maintenance of salamander skin bacterial communities. If deprived of a natural soil reservoir, the bacterial communities on the skin of salamanders greatly changed. In particular, the richness and evenness of bacterial communities greatly decreased for salamanders without a reservoir. Furthermore, we found a core community consisting of a dominant Verrucomicrobia OTU, one Staphylococcaceae OTU, one Comamonadaceae OTU, and five Pseudomonadaceae OTUs (Loudon et al., 2014b). These findings led us to ask whether neutral or non-neutral processes were important in maintaining these communities.

We experimentally housed salamanders with or without a bacterial reservoir (i.e., soil) and determined how bacterial reservoirs affected the dispersion (i.e., over, neutral, or under-represented) and the potential function of bacterial OTUs on salamanders’ skin. Furthermore, we closely examined neutral processes by characterizing immigration and loss (emigration and local extinction) rates and persistence of bacterial OTUs from our neutral model. We hypothesize that anti-*B. dendrobatidis* OTUs are over-represented on salamanders compared to soil, and that anti-*B. dendrobatidis* OTUs are more likely to persist on salamanders.

## Materials and Methods

### Experimental Design and Molecular Methods

Methods regarding salamander capture, housing, rearing conditions, and molecular characterization of cutaneous microbial communities are given in detail in Loudon et al. (2014b). In brief, red-backed salamanders, *P. cinereus*, were collected from George Washington National Forest in October 2011. Twenty salamanders were collected in total, and initial skin swabs and soil samples closest to each salamander were taken in the field. Salamanders were assigned at random to laboratory containers with natural soil collected from the salamanders’ habitat or with sterile water (i.e., with or without a bacterial reservoir; *N* = 10 per treatment). These salamanders were maintained for 4 weeks in the lab and sampled weekly. Prior to sampling, salamanders were rinsed with sterile water three times to remove loosely adhered bacteria (Culp et al., 2007; Lauer et al., 2007). Soil was collected from the same site as the salamanders and then taken to the lab. Initial samples were taken from this soil in triplicate; the soil was then distributed to their respective laboratory containers and subsequently sampled in triplicate on days 14 and 28. This sampling scheme allowed us to compare the bacterial communities on salamanders directly to their respective soil bacterial communities, if soil was present. Bacterial communities were assessed by sequencing the V4 region of the 16S rRNA gene and yielded a 100 bp product. Sequencing was performed using the Illumina HiSeq platform with sample prep, primers, and PCR and conducted according to standard practice (Caporaso et al., 2010c, 2012). Clustering of sequences into OTUs, and subsequent bioinformatic analysis was done using QIIME 1.7.0 (Caporaso et al., 2010b). 23.3 million sequences met stringent quality control thresholds and were included for ‘open-reference’ OTU picking (Navas-Molina et al., 2013). In brief, open-reference picking combines the standard ‘closed-reference’ approach with clustering of novel sequences that do not match the reference collection at a similarity of 97% sequence identity. Of the reads, 83% hit the reference data set and were assigned a reference GreenGenes taxonomy (McDonald et al., 2012). The remaining sequences were clustered into *de novo* OTUs, and taxonomy was assigned using the RDP classifier (Wang et al., 2007) that was retrained on the GreenGenes 2012 data with an 80% confidence threshold. All reads were clustered at 97% similarity using USEARCH (Edgar, 2010). OTUs with less than 100 reads were discarded (Bokulich et al., 2013), which resulted in 21.7 million sequences and a total of 6049 OTUs. PyNAST (DeSantis et al., 2006a; Caporaso et al., 2010a) was used to align the sequences to the GreenGenes reference and a phylogenetic tree was constructed using FastTree (Price et al., 2010). Prior to analysis, the OTU table was rarefied at 34,000 sequences per sample. This level balanced the removal of low coverage samples with removal of sequences from high coverage samples. Exploratory, analyses were also conducted at higher and lower levels of rarefaction, and patterns found were not different.

These data, along with MiMARKs compliant metadata ^1^, are available in the Quantitative Insights Into Microbial Ecology (QIIME) database ^2^; study no. 1618). Data have been deposited at the European Bioinformatics Institute (EBI) archive with the accession number ERP003771.

### Neutral Model

**FIGURE 1:**
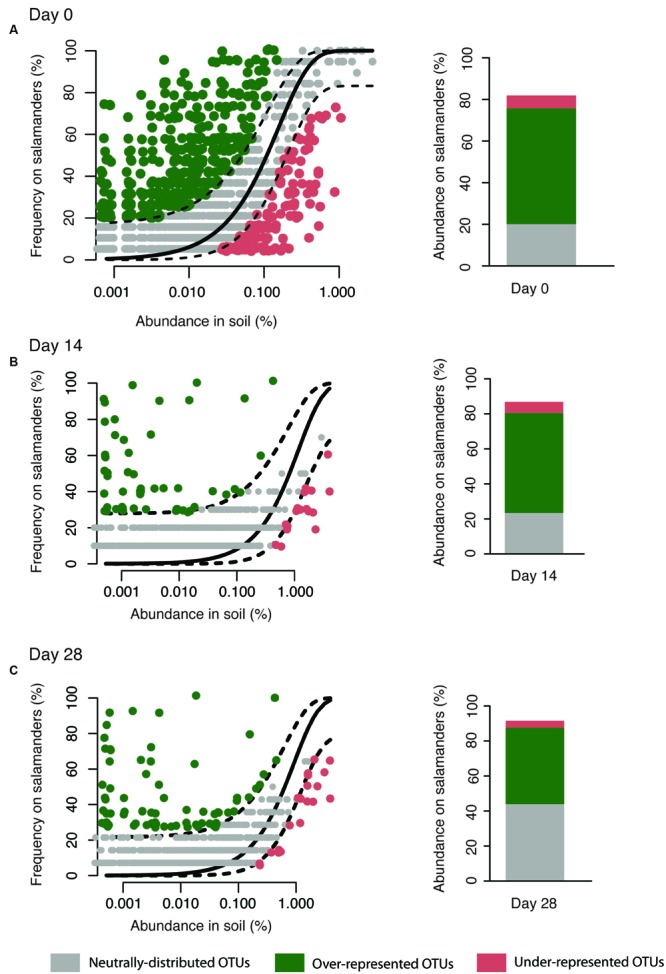
**Neutral model applied to the salamander skin microbiota with soil as the source.** Stacked bar chart depicts the relative abundance of sequences in the neutrally-distributed, over-represented, and under-represented OTUs on salamander skin. Since, the neutral model can be applied only to OTUs shared between the salamander and soil, the abundances do not add up to 100% in the bar chart. The model was applied independently on days 0 **(A)**, 14 **(B)**, and 28 **(C)**.

Our aim was to assess the relative importance of neutral (random dispersal from soil and ecological drift) and selective processes in maintaining the composition of the salamander skin bacterial community. We used a neutral model of community ecology for this purpose, and considered the bacterial community in soil as the source of the bacteria on salamander skins. Only salamanders housed with soil (and not the no reservoir treatment) were included in this analysis since it more realistically mimics the conditions of wild salamanders that would be in constant contact with environmental bacteria, and because this analysis requires a source community. The model (Sloan et al., 2006; Morris et al., 2013; Venkataraman et al., 2015) was applied independently at three time points: at day of salamander capture (day 0) and after 14 and 28 days of laboratory housing (days 14 and 28). The basic mathematical premise of our neutral community model is that the probability of detecting an OTU on salamander skin due to random dispersal and ecological drift is directly proportional to the abundance of that OTU in the source community – soil. The relative abundance of an OTU in soil is calculated by dividing the number of 16S-rRNA encoding gene sequences in that OTU by the total number of 16S-sequences recovered from soil. The observed probability of detection for each OTU on salamander skins is calculated as the fraction of salamanders on whom that OTU is detected (20 wild salamanders surveyed on day 0 here; if detected on just one salamander the observed detection probability is 5% and if detected on all 20 salamanders it is 100%). Finally, a beta-probability distribution is used to predict the expected probability of detection of an OTU if it was present on salamander skins via dispersal limitation and ecological drift. The inputs into this probability distribution are an overall fitting parameter (N_t_ × m) and the relative abundance of the OTU in soil. N_t_ is the total community size and m denotes the probability of dispersal of an organism (OTU here) from soil to salamander skins (Hubbell, 2001). 16S-surveys typically provide relative abundances rather than absolute abundances, thus it is not possible to get accurate estimates of N_t_. Hence in this neutral model, N_t_ and m are lumped together as a fitting parameter N_t_m. The value of this parameter is optimized using a least-squares approach such that the sum of squares of residuals between the observed and predicted detection probability for all OTUs is minimized. The variability around this predicted detection frequency is calculated using 95% binomial proportion confidence intervals (Wilson method) with the HMisc package in R. OTUs whose observed detection frequency falls within the 95% confidence intervals of the predicted detection frequency are identified as being consistent with the neutral model, suggesting that their presence on salamander skins is consistent with dispersal from soil and ecological drift on the salamander (gray points in **Figure 1**). OTUs positively deviating from the model expectations are over-represented on the salamander skins (green points in **Figure 1**). These are the strongest candidates for having a competitive advantage, being resistant to host filters [i.e., antimicrobial peptides (AMPs)], or better at dispersal relative to other bacteria in the soil. The negatively deviating OTUs are under-represented on salamander skins (red points in **Figure 1**). These can be interpreted as being selected against by the salamander, being poor competitors, susceptible to host filters (i.e., AMPs secreted by the host), or limited with respect to dispersal from soil (e.g., present only in center of large soil aggregates). Finally, the cumulative relative abundance of sequences in these three categories of OTUs on salamander skins is used as a proxy to evaluate the contributions of neutral processes (Σ *Rel.abund.of sequences within grey OTUs*) and selection (Σ *Rel.abund.of sequences in green and red OTUs*) in shaping the skin microbiota.

### Proportion of Fungal Inhibitory OTUs

A number of studies have cultured bacteria isolated from amphibian skin and tested these isolates for bioactivity against the amphibian fungal pathogen *B. dendrobatidis* in co-culture challenge assays (Harris et al., 2006; Lauer et al., 2007, 2008; Woodhams et al., 2007; Lam et al., 2010; Walke et al., 2011; Flechas et al., 2012; Bell et al., 2013; Woodhams et al., 2015). Woodhams et al. (2015) generated an antifungal database by compiling the 1255 16S rRNA gene sequences from bacteria isolated from amphibians’ skin; 819 of these isolates inhibited *B. dendrobatidis* in challenge assays. From this database, we generated a list of OTUs by clustering sequences at ≥97% similarity using the Green Genes October 2012 reference (DeSantis et al., 2006b), which is the same criterion we used to create OTUs in our salamander skin 16S-surveys. The full Sanger sequences (mean 1074.8 bp) of isolates that inhibited *B. dendrobatidis* growth were trimmed to the first 100 bp beyond primer 515f to overlap with Illumina reads. The 819 anti-*B. dendrobatidis* isolates clustered into 327 OTUs at ≥97% similarity. We then asked how many of the OTUs categorized as “over-represented,” “under-represented,” and “neutrally-distributed” in the neutral-model analysis had a 97% similarity to these 819 anti-*B. dendrobatidis* isolates. For this analysis to be most robust, we focused on OTUs that consistently fell into the same category (i.e., over-represented, neutrally-distributed, under-represented) for all three time points were examined. The proportions of anti-*B. dendrobatidis* OTUs in each of these three categories were compared by randomization test of independence with 100,000 iterations. In addition, using Geneious version 7.1.7 (Biomatters, Ltd., Auckland, New Zealand), we found the highest matches in the database to potential non-antifungal or enhanced fungal growth isolates.

### Immigration and Loss Rates

To determine how immigration and loss changes in the presence absence of a bacterial reservoir we calculated immigration and loss rates. This was done for each individual salamander: we isolated the samples from each salamander, and calculated OTU gains and losses between time points. Specifically, we created ordered pairs (x,y) with x unique taxa at time n, and y the gain or loss of OTUs between time n and n+1. Gain was calculated as number of unique taxa at time point n+1 not found at time point n, and loss number of unique taxa found at time point n not found at time point n+1. Salamander samples were segregated prior to analysis by treatment (presence or absence of bacteria reservoir) and regression coefficients were calculated for immigration and loss (emigration and local extinction) using linear least squares.

### Persistence of Antifungal and Non-antifungal OTUs

To determine if persistent bacteria were more likely to be related to antifungal bacterial isolates we assigned each OTU a persistence score. The number of times a given OTU was found on at least one salamander was scored on a scale of 5 per sample (present at all five time points) to 0 (never present) and was calculated for all salamanders within a treatment through time. The resulting scores were compared between known antifungal OTUs and OTUs not known to be antifungal (after accounting for salamander treatment differences) using a Mann–Whitney *U*-test. These methods may underestimate antifungal OTUs, as some OTUs may be inhibitory but not previously characterized by other studies. Conversely, some bacteria that are classified as inhibitory may be non-inhibitory under other environmental conditions (Woodhams et al., 2014).

### Persistence of Over and Under-represented OTUs

To link our neutral analysis to the persistence of bacteria we determined the persistence score for the OTUs that consistently fell into either the over-represented or under-represented category from both of our treatments (i.e., with or without a bacterial reservoir). A Welch’s *t*-test was used to compare the means of the persistence scores for each treatment.

## Results

We used a neutral model (**Figure 1**) to analyze the 3058 OTUs that were shared between salamander skin and soil; this model only incorporates shared OTUs. These OTUs cumulatively made up to 92% of the 16S-sequences on salamanders’ skin. The model was applied on data derived from days 14 to 28 of housing as well. Across all three time points (days 0, 14, and 28), we found that up to 39% of the sequences recovered from salamander skin fell into OTUs consistent with neutral-distribution (gray points in **Figure 1**), whereas up to 58% of the sequences fell into OTUs that were over-represented on the salamander skins (green points in **Figure 1**), and up to 6% of the sequences fall into under-represented OTUs (red points in **Figure 1**). We focused on bacterial OTUs that fell into the over, under and neutrally distributed bins consistently through the experiment, i.e., on days 0, 14, and 28; this consistency allowed us to be more confident of our classifications. Overall, 272 OTUs were consistently neutrally-distributed at all three time points, 12 OTUs were consistently over-represented, and 9 were consistently under-represented OTUs (**Table 1**). In terms in temporal variability, on day 28, the relative abundance of sequences from OTUs that were neutral increased compared to days 0 and 14 (**Figure 1**).

**Table 1:** The number of operational taxonomic units (OTUs) on salamanders that were consistently over-represented, neutrally-represented, or under-represented and the number of each that matches antifungal isolates between all three time points.

OTU status	Total OTUs	Antifungal OTUs
Over-represented	12	9
Neutrally-represented	272	2
Under-represented	9	0

To measure the prevalence of antifungal OTUs in these three categories, we determined which OTUs were within 97% sequence similarity to antifungal bacterial isolates in a curated database of the 16S rRNA gene sequence of bacterial isolates known to inhibit the pathogen, *B. dendrobatidis* (Woodhams et al., 2015). Our analysis revealed that out of the 272 neutrally-distributed OTUs, only two (0.7%) match antifungal isolates (**Table 1**). Of the nine OTUs that were always under-represented on the host (Supplementary Table S1), none had a 97% or greater similarity match to antifungal isolates. As expected conceptually, of the 12 OTUs that were always over-represented on the host, nine OTUs (75%) had a 97% or greater similarity match to previously identified antifungal isolates (**Tables 1** and **2**). These OTUs can also have the same percent match to isolates that do not exhibit antifungal activity or enhance fungal growth (**Table 2**). Using a randomized test of independence with 100,000 iterations, we confirmed that this differential distribution of OTUs similar to antifungal isolates in the three categories of the neutral model analysis was statistically significant (*P* < 0.0001). Among the over-represented OTUs, four are considered to be in the salamanders’ ‘core community’ (**Table 2**), defined as being present on 90% or more of all salamanders through time (Loudon et al., 2014b). The relative abundance of these OTUs on wild salamanders varied between 0.01 and 4.29%. The relative abundance of the over-abundant OTUs combined was 7.9% ± 11.9 SD.

**Table 2:** The taxonomy, functional match status, prevalence, relative abundance, and core community status (determined in Loudon et al., 2014b) of OTUs that were over-represented on salamanders using the neutral model.

Greengenes OTU #	Taxonomy	Antifungal match?	Non-antifungal match	Enhancing match	Percent nucleotide match	Prevalence on wild salamanders (*n* = 19)	Relative abundance (%) and SD on wild salamanders	Member of core community (Loudon et al., 2014b)
164589	Opitutuae	Unknown	Unknown	Unknown	Unknown	0.74	0.14 ± 0.22	Yes
1121948	Sphingobacteriaceae	Unknown	Unknown	Unknown	Unknown	0.21	0.01 ± 0.02	No
166553	Bradyrhizobiaceae; *Bosea*	Unknown	Unknown	Unknown	Unknown	0.89	0.19 ± 0.21	No
573223	Rhizobiaceae; *Rhizobium*	Yes	No	No	100	0.5	0.08 ± 0.14	No
351280	Oxalobacteraceae; *Janthinobacterium lividum*	Yes	Yes	No	100	0.95	0.58 ± 0.96	No
321153	Oxalobacteraceae; *Janthinobacterium lividum*	Yes	Yes	No	100	0.26	0.01 ± 0.02	No
292134	Enterobacteriaceae	Yes	Yes	Yes	100	0.58	0.14 ± 0.28	No
279948	Pseudomonadaceae	Yes	Yes	Yes	100	1	4.29 ± 9.66	Yes
144755	Pseudomonadaceae	Yes	Yes	Yes	100	0.95	1.53 ± 2.22	Yes
293741	Pseudomonadaceae; *Pseudomonas*	Yes	No	No	100	1	0.55 ± 0.97	No
825181	Pseudomonadaceae; *Pseudomonas*	Yes	Yes	Yes	99	0.79	0.23 ± 0.52	Yes
561294	Pseudomonadaceae; *Pseudomonas*	Yes	Yes	Yes	100	0.68	0.16 ± 0.25	No

*Percent nucleotide match is the same for all functional categories.*

**FIGURE 2:**
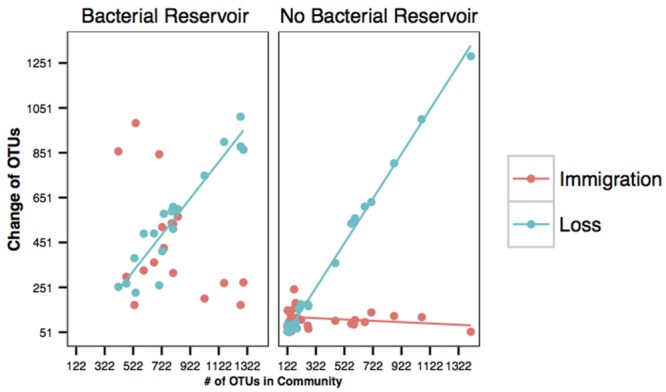
**Immigration (salmon) and loss (blue) curves of bacteria on amphibians’ skin.** Each point represents one salamander at any given time throughout the experiment. Change represents both gain and loss. For example, points denoted as loss means that y OTUs were lost. Immigration (salmon) and loss (blue) curves with a bacterial reservoir. Immigration rate (*r*^2^ = 0.063, β_1_ = -0.233) was not related to species richness, but extinction rate (*r*^2^ = 0.886, β_1_ = 0.812) was related to species richness. Immigration (salmon) and loss (blue) curves in the absence of a bacterial reservoir. Immigration rate (*r*^2^ = 0.06, β_1_ = -0.03) was not related to species richness, but loss rate (*r*^2^ = 0.995, β_1_ = 0.994) was related to species richness.

The immigration of OTUs not already found on the skin was not a function of species richness on amphibian skins in either the with or without bacterial reservoir treatments (**Figure 2**). As expected in the sterile environment (without a bacterial reservoir), there was no change in the immigration rate of new taxa over time because the only microbes in the system came in on the salamanders (**Figure 2**). However, the local extinction rate was strongly positively correlated with species richness on amphibian skins in both environments (**Figure 2**).

Operational taxonomic units that had a 97% or greater match to antifungal isolates were significantly more likely to be found at more time points than OTUs not known to be antifungal for salamanders with a bacterial reservoir (*U* = 590473.5, *P* < 0.001). This result was the same for salamanders housed without a bacterial reservoir (*U* = 363500.0, *P* < 0.001).

**FIGURE 3:**
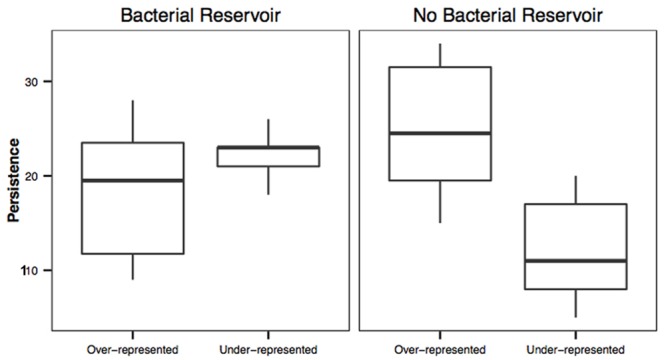
**Persistence of over-represented and under-represented OTUs on salamanders with or without a bacterial reservoir.** Over and under-represented OTUs on salamanders with a bacterial reservoir had similar persistence on salamanders (*t* = -1.65, df = 15.01, *P* = 0.119), whereas under-represented OTUs were less persistent on salamanders without a bacterial reservoir (*t* = -4.55, df = 18.9, *P* < 0.001).

Over-represented OTUs were more persistent on salamanders housed without a bacterial reservoir than under-represented OTUs (*t* = -4.55, df = 18.9, *P* < 0.001; **Figure 3**). However, there was no difference in persistence between over-represented and under-represented OTUs on salamanders housed with a bacterial reservoir (*t* = -1.65, df = 15.01, *P* = 0.119; **Figure 3**).

## Discussion

In this study, we modeled the relative contributions of neutral processes (random dispersal from soil and ecological drift), and selective processes (interspecies competition, selection by the host, exposure to *B. dendrobatidis*) in shaping the composition of the salamander’s skin bacteria. For this purpose, we applied a neutral community model and compared the distribution of OTUs between soil and salamander skins at three time points (days 0, 14, and 28). A majority of sequences (up to 58%) at all time points from salamander skins fell into over-represented OTUs, while neutrally-distributed OTUs account for up to 39% of the sequences. Therefore, the neutral model analyses are consistent with both selection and neutral-processes contributing to maintaining the salamander skin bacteria, with selection by the host playing a larger role in this habitat. Furthermore, we show that only a few OTUs on the salamanders comprised a large proportion of the community and this is consistent with the hypothesis that the over-represented bacteria are being selected for and enriched on salamander’s skin.

The relative abundance of sequences from neutral OTUs increased while sequences from over-represented OTUs decreased on day 28 (**Figure 1**). The observation on day 28 may be due to the length of time that the salamanders and soil had been in the laboratory, with results deviating from the conditions in nature over time. This may be a trend, but without further long-term data, this possibility cannot be evaluated. Alternatively, this increase on day 28 may be explained if there was stochastic variation over time in the proportion of sequences that are neutrally distributed, over-represented, and under-represented. For example one of the neutral OTUs may have bloomed during the time of our sampling.

Four of the 12 OTUs that were consistently over-represented on the salamanders were in the salamander ‘core community’ (**Table 2**), which we define as OTUs found on 90% or more on salamanders at all time points and in field and laboratory conditions (Loudon et al., 2014b). There were also four OTUs in the ‘core community’ that were identified as neutrally-distributed, because they were also present in high relative abundance in the soil (**Table 2**). As such the neutral model analysis does not take into account nor provide any information regarding the functional potential of microbes. It is possible that some of these neutrally-distributed OTUs serve a function on salamander skins by for example promoting interference competition, but establishing this requires further experimentation. These consistently over-represented OTUs had an average relative abundance of 7.9% ± 11.9 on wild salamanders, and are amongst the most abundant OTUs found on salamanders. For example, the most relatively abundant OTU on wild salamanders, over-represented OTU 279948, consisted on average of 4.29% of the community (**Table 2**).

There are multiple potential explanations for why some bacteria are under-represented on salamander skin. Some OTUs could be detrimental to the hosts, and salamanders may have evolved active mechanisms, such as AMP secretions, to deter persistence of these OTUs despite constant contact with the surrounding soil. Some resident skin bacteria may inhibit colonization or out compete some soil bacteria. For example, 89% of the consistently under-represented bacteria belong to the phylum *Acidobacteria*. Members of this phylum are typically slow growing soil bacteria that thrive in oligotrophic conditions (Ward et al., 2009) and may be poor competitors on the relatively copiotrophic salamander skin, which is covered with mucus, due to their slow growth rate.

Skin infection by the fungal pathogen *B. dendrobatidis* is one of the leading causes of amphibian mortality, global population declines, and extinctions (Skerratt et al., 2007). Since a fungal infection by *B. dendrobatidis* and other fungi (Banning et al., 2008) is likely a strong selective pressure on amphibians and their embryos, it is conceivable that the selection for antifungal bacterial species on amphibians’ skin has occurred. The host species in this study has likely been exposed to *B. dendrobatidis* for at least a century, since *B. dendrobatidis* was earliest observed in North American museum specimens in 1888 (Talley et al., 2015); thus ample time for selection has occurred. Accordingly, we found that sequences in 75% of the OTUs that were consistently over-represented on salamanders closely matched known antifungal isolates (within 97% or greater sequence similarity) whereas exceedingly few of the neutrally-distributed OTUs (0.7%) and none of the under-represented OTUs had a 97% or greater match to antifungal isolates. It is important to note that these matches to the antifungal database do not necessarily mean that these bacteria exhibit antifungal activity, but that they are strong candidates for exhibiting antifungal activity. Indeed, these over-represented OTUs can also match isolates found on amphibians’ skin that did not exhibit antifungal activity or even enhance fungal growth. Culturing the specific isolates that are over-represented and testing their ability to inhibit fungi would be an excellent follow-up study. However, our results suggest that the salamander skin may actively select for bacterial species with antifungal activity. Our central result remains that over-represented OTUs match antifungal isolates whereas neutral and under-represented OTUs do not.

The selection of beneficial bacterial communities may occur without hosts selecting for specific OTUs if they maintain conditions on the skin that are favorable to defensive microbes (Scheuring and Yu, 2012). The selected antifungal bacteria included *J. lividum* and members of Enterobacteriaceae and Pseudomonadaceae (**Table 2**), which are thought to play large roles in disease protection (Becker et al., 2009, 2015; Harris et al., 2009). It should be noted that the antifungal database consists of bacteria that are cultured, and therefore is biased toward culturable species, although there is no reason to think antifungal species would be any less represented among uncultured species.

Operational taxonomic units that have a high similarity to antifungal isolates were more likely to persist on salamanders than OTUs that were not known to be antifungal. This links the extinction curve analysis from island biogeography to the neutral model analysis by suggesting that the OTUs that do not go extinct or emigrate are antifungal and are likely to be selected to persist on the salamander skins. Furthermore, we determined that persistence of OTUs between over-represented and under-represented OTUs differed between our two treatments. There was no difference in persistence score between over and under-represented OTUs for salamanders with a bacterial reservoir when antifungal status is not considered. This is likely because the salamanders in soil are continually in contact with the environmental bacteria that are likely passively immigrating onto the salamanders, including under-represented OTUs, and therefore there would be a greater chance of detecting transient OTUs through time. However, in the absence of a bacterial reservoir, the immigration of environmental bacteria is greatly reduced (as demonstrated in **Figure 2**) and the persistence of under-represented OTUs is reduced. These results demonstrate that the under-represented OTUs need a reservoir in order to be detected on salamanders and further suggests that bacteria are selected for and against on salamanders.

The results from our immigration and loss analyses demonstrate that the principles of island biogeography apply the skins of salamanders. The principles of island biogeography have provided insights into the maintenance of host-associated bacterial communities previously, although these principles have rarely been applied in this context or to microbial communities in general (Horner-Devine et al., 2004; Bell et al., 2005; Fierer, 2008; Johnson and Winquist, 2011; Nemergut et al., 2011; Bassis et al., 2015). We also experimentally determined how immigration and loss are affected by a bacterial reservoir. In this study, immigration rate was not significantly related to species richness, whereas loss rate was strongly related to richness. These results suggest that microbes are invading salamanders without respect to the species richness, but persisting on salamanders is strongly related to richness. Communities that have a greater richness appear to be saturated, thereby leading to an increased extinction or loss rate. This relationship suggests that interspecific competition is likely a force that structures amphibians’ cutaneous communities. Indeed, persistent bacteria were more likely to be antifungal, which may be a result of their ability to succeed in interspecific competition. This hypothesis could be tested by experimentally lowering richness using an antibiotic bath (Becker and Harris, 2010) and determining if loss rate is lowered. Complementary experiments could be conducted by increasing richness on the skin and assaying extinction rate; these experiments should be conducted in a system where immigration from a soil reservoir is not possible, e.g., sterile water (Loudon et al., 2014b), and therefore extinctions are less likely to be missed.

Our findings link bacteria under positive selection with antifungal activity and persistence on salamanders. Our emerging hypothesis is that soil microbes freely colonize salamander skins and are part of the transient species pool, but the OTUs adapted to amphibian skins are the ones that have a competitive advantage and persist. The competitive advantage of some bacteria is increased by their production for antifungal metabolites (Loudon et al., 2014a), which protect the host from fungal diseases as a by-product. This research will aid future research in using bioaugmentation as a conservation strategy against disease (Bletz et al., 2013). Knowing which bacteria are selected for and have the ability to persist on a host is vital when selecting probiotic candidates.

Lastly, examining the known antifungal bacteria within salamanders’ bacterial communities may be a helpful approach in assessing the risk of North American salamanders to the recently described pathogen *B. salamandrivorans*, which is found in Europe (Martel et al., 2013). Currently the pathogen, which is more virulent to salamanders than to frogs and toads (Martel et al., 2014), has not been detected in North America (Muletz et al., 2014; Bales et al., 2015), but is likely to arrive.

## Author Contributions

All authors contributed to the conceptual framework of the paper. AL and RH designed and executed the experiment. AV performed the neutral model analysis, and WV performed island biogeography analysis. AL, RH, AV, WV wrote the manuscript. All authors contributed substantially to the revisions.

## Conflict of Interest Statement

The authors declare that the research was conducted in the absence of any commercial or financial relationships that could be construed as a potential conflict of interest.

## Footnotes

1 http://gensc.org/gc_wiki/index.php/MIMARKS

2 www.microbio.me/qiime
