# Supplementary material for: Vertebrate Hosts as Islands: Dynamics of Selection, Immigration, Loss, Persistence, and Potential Function of Bacteria on Salamander Skin
Source: Front Microbiol. 2016 Mar 16;7:333. doi: 10.3389/fmicb.2016.00333 (PMC4793798; doi:10.3389/fmicb.2016.00333)
Supplement: Supplementary file 1 [file Table_1.PDF]

Supplementary table 1. Taxonomy of OTUs that were under-selected on salamanders using the neutral model.

Greenegenes OTU #	Class	Order	Family
1136012	Acidobacteria	Acidobacteriales	Koribacteraceae
512382	Acidobacteria	Acidobacteriales	Koribacteraceae
280477	Acidobacteria	Acidobacteriales	Koribacteraceae
114255	Acidobacteria	Acidobacteriales	Koribacteraceae
563862	Acidobacteria	Unknown	Unknown
46444	Acidobacteria	Unknown	Unknown
113212	Acidobacteria	Unknown	Unknown
153088	Acidobacteria	Unknown	Unknown
252578	Deltaproteobacteria	Syntrophobacterales	Syntrophobacteraceae
